# Asthma, Its Species and Complications

**Published:** 1835-07-01

**Authors:** 


*Asthma, its Species and Complications. Illustrated by Cases, and Plates coloured from Nature.* By FRANCIS HOPKINS RAMADGE, M. D., &c. London, 1835. 8vo. pp. 360.

"THERE is no book so bad," quoth an optimist author, "but that something may be learned from it." This is strangely exemplified in the present instance. The lesson to be derived from it, however, is not medical, but moral, and relates to the conduct of life, not to the curing of asthmas. The young student will learn from Dr. Ramadge's book, far better than if it were a professed lecture against carping, how dangerous it is for a man of mediocre powers and attainments to separate himself from the rest of his brethren, to snarl alike at the successful living and the illustrious dead; and, without the energies of a son of the desert, to enact the Esau, with his hand against every man, and every man's hand against his.

Seen through the jaundiced medium of Dr. Ramadge's misanthropy, Heberden was a superficial fellow; Dr. Bree's pathological views are meagre and irrelevant; Dr. Withers was a remarkably mistaken man; Baillie no anatomist; Radcliffe an empiric; Dr. Latham was not industrious; and Sir Henry Halford, says our unfortunate author, is quite unfit for the place of President of the College of Physicians. But our readers will enjoy these oddities in an unabbreviated form. Here are a few of them.

"Perhaps, however, we ought to charge this want of knowledge on his 'most respected preceptor,' the elder Latham, whom he compliments with a degree of eleemosynary gratitude. It was doubtless charitable and generous in him so to do; since it is to be presumed from Dr. Latham's work on Diabetes, as well as his holding a situation in a hospital, to an appointment in which none are eligible who have not derived their medical, or, more correctly speaking, non-medical knowledge from the old Universities, that his pupils could not have been over and above enlightened by the worthy doctor's industrious efforts. Industry, indeed, like every other word, has its signification limited by the ideas, and habits, of the person pronouncing it.

"In the work above cited on Diabetes, Dr. Latham boasts of retiring on the well-earned fruits of industry: and, as a specimen of his notions on the subject of industry, he writes a treatise on the complaint after having barely investigated 'a case or two' by dissection. This he deemed enough, and, I presume, felt fatigued by the exertion. In addition to this, he had doubtless imbibed a distaste to morbid anatomy from the system of education pursued at Oxford and Cambridge." (P. 32.)

"I have quoted these instances from Heberden, as I probably shall do from others, rather with a view of showing that I have not neglected the writings of those whom, as a member of the College of Physicians, I am bound to reverence, than for any high opinion of their science. A note-book like Heberden's proves his attention to appearances, but throws no light on causes. However, like the cabinet of the virtuoso, the curiosities the possessor cannot explain, others may. I allude to the species of writing, not to Heberden. His fame was well-earned, for he wrote in Latin." (P. 58.)

"Dr. Bree gives us many such; and what he relates, as coming under his own experience, is far from unexceptionable. Indeed, his pathological views are altogether meagre and irrelevant; those cases, which he adduces as belonging to the uncomplicated form, being merely symptomatic." (P. 96.)

"This same Galen of the North [Dr. Withers] gives an amusing specimen of his skill in conducting pathological enquiry, in the examination of the body of one John Strickney. All that he appears to have found was water, water in the abdomen, water in the chest, water in the pericardium; and, had he opened the cranium, he would doubtless have found water there also!

"On the whole, he appears to have been just as fit for the situation he held of Physician to a County Hospital, as many of our metropolitan ones, (i. e. up to a very recent period,) who, whatever else they might do, abstained as religiously from meddling with a dead body as if they were converts to the Hebrew faith." (P. 100.)

"Indeed, an œdematous state of the lungs, which both Doctors Baillie and Blackall consider of uncommon occurrence, is, on the contrary, very frequently seen on dissection. Scarcely an individual dies of chronic disease, with swelled extremities, whose lungs are not more or less œdematous. I fear the first gentleman derived what he knew of morbid anatomy from inspecting the museum of his uncle, Dr. Hunter, rather than from observations made on the human body: a bad method for the greatest morbid anatomist our country has produced, to pursue, but one well-calculated to induce his ignorance of that anasarcous condition so generally recognized by the practical pathologist." (P. 113.)

"Having alluded to the case of the above monarch, I may still further observe that he was troubled with an unusual quantity of rheum or phlegm, and it was to lessen this that Radcliffe directed his attention; a successful empiric, whose gold-headed cane, after having passed through the hands of several other physicians, to whom the old proverb, 'great cry and little wool,' may be fairly applied, since they all enjoy celebrity, without having effected much to forward the interests of science, was not long ago deposited in the library of the College of Physicians. From this censure I must except the last, Dr. Baillie; who, if he had examined the human body with the same attention he devoted to the preparations of his uncle Dr. Hunter, might have produced something worthy of posterity." (P. 194.)

*Source of Dr. Armstrong's errors.* "Different balsams have been lauded in this disease, and especially that vulgarly called the balsam of copaiba. This has met with several admirers, and the late Dr. Armstrong, in particular, has been loud in its praise. He wrote, however, I apprehend, from views chiefly gleaned in private practice; and such must ever labour under the defects inseparable from insufficient data. Copaiba is usually very revolting to the stomach, and is far from being certain either to soothe the cough, or alter the expectoration." (P. 251.)

*Professional humanity.* "So easy, indeed, is it of proof to the morbid anatomist, that although it seems surprising to me it should have escaped the activity of modern research, previously to my recognition of the fact; yet, when once pointed out, I cannot help thinking that to overlook it argues a blindness as complete as that of the Homeric Cyclops. I was about to say that this mental darkness was unaccountable; but to him, who has gained experience in humanity, professional humanity, it is no riddle." (P. 346.)

At p. 235, *et seq.* there is an important, and almost miraculous case, which we must endeavour to abridge. Dr. Ramadge thus begins the history.

"Having the privilege of visiting and witnessing the medical practice of the —— Hospital, in virtue of a sum of money, for which I cannot say I ever had value received, I was, in flattering terms, requested by one of the physicians, in the presence of some of his pupils, to give him my opinion of a patient, who was suffering under catarrhal asthma, with pituitous discharge to a great extent." (P. 235.)

Our author told the physician of Blank Hospital that he was treating the patient quite erroneously. Though this salutary rebuke was no doubt expressed in the dulcet strain, and with the hushing gentility with which we have seen that Dr. Ramadge deals forth his aphorisms, the mistaken doctor, "who was indebted for his situation, not so much to his talent, as to his happy stars," changed his compliments "into affected contempt." The patient, of course, got worse and worse, withdrew from Blank Hospital, barely alive, and applied to our author.

"When I saw him at this period, his countenance was bloated, anxious, sub-livid; his lips, nose, eminences of the cheeks, and lobes of the ears, were perfectly blue; his breathing high and painfully laborious; he could with difficulty support himself on his legs, which were quite dropsical. His cough and expectoration were almost incessant. He looked as if he had been long a stranger to sleep; and I found, indeed, that the dread of suffocation prevented his giving way to 'tired nature's sweet restorer;' and that, when exhausted by sufferings and watching, he snatched a few minutes' repose, he would suddenly be awakened by the most fearful dreams." (P. 237.)

Putting physicians who owe their situations to their "happy stars" out of the question, we should think that even a good average doctor would scarcely be able to keep a moribund of this kind alive for forty-eight hours. Not so Dr. Ramadge: he cured the man perfectly; told him how to secure himself against a relapse, and, above all, enjoined him to be on his guard "against the ingression of cold." Nor was this all: "I dismissed him with a recommendation to show himself to his former medical curator at the —— Hospital. I could do no less in return for the urbanity with which that person had treated me."

Why have we no record of the interview between the man of happy stars and his resuscitated patient? the doctor must have looked as blank as his hospital.

A trial however is recorded at p. 303, compared to which this case, in our humble opinion, is a mere fleabite. A banker having died suddenly, the Rock Insurance Office refused to pay their policy, on the plea that the deceased had committed suicide. More than one hospital surgeon gave it as his opinion that he had taken poison. And how do you suppose, L. B., that the question was settled the other way? Was Dr. Ramadge obliged to go down, and read the jury a lecture on imaginary poisoning? By no means: a *Deus ex machinâ* is, after all, a clumsy mode of solving difficulties. A former pupil of the doctor's did the job, and as juries do not number but weigh witnesses, the hospital surgeons kicked the beam, and the executors pouched the cash.

The hasty reader, who skips and skims, and, arriving at p. 310, finds the running title to be still "Asthma complicated with organic lesions of the heart, &c." must not suppose that the page is really occupied with so uninteresting a subject: it is the evening meetings at the College of Physicians, and the fitness of Sir H. Halford to be president, which are discussed in that and several subsequent pages. Thus, our Timon tells us that "such meetings may tend to introduce some half-dozen sucking favourites of the Galenical Sultan's to promising patients, and gratify personal vanity at the same time. The non-professional hearer will take oratorical common-places for the dicta of an Hippocrates, and the initiated will be wiser than to gainsay his επεα πτεροεντα." (P. 312.)

We must request the College, however, not to take all this too much to heart: our misiatrous author was only joking. The grave and heart-rending part comes directly afterwards: "But, seriously speaking, is this a state of things that ought to exist," &c. You see, gentle reader, how pleasantly our misanthrope can relax; how graciously he smiles, like Apollo with his arrows thrown away. How elegantly he applies an old adage! "The old proverb, which, in its Latin dress, "*Quid nocet alteri, alteri juvat,*" does not look amiss; but which, in its English garb, is not quite the thing for 'ears polite,' although equally as true,—to wit, 'what is one man's meat is another's poison,' was never more fully verified than in this complaint." (P. 171.)

We will now claim our author's privilege of "seriously speaking," and dismiss him with the following advice: Let him go on in the quiet, unknown way, that he did five or six years ago: let him give up his foolish quarrels with the profession, suffer his books to sink into oblivion, (i. e. cease to advertise them,) and, above all, renounce his attempts at criticism. For this last office he is so especially disqualified, that we venture to say, he will be hit twenty times before he hits once. *Lepus tute es, et pulpamentum quæris?*

